# Fayez never saw sunlight: There is a need for more specialized respiratory units

**DOI:** 10.4103/1817-1737.44777

**Published:** 2009

**Authors:** Abdullah Al Mobeireek

**Affiliations:** *Pulmonary Section, Department of Medicine, King Faisal Specialist Hospital and Research Center, Riyadh, Saudi Arabia*

Fayez was a 16-year-old boy, although he was so small that he appeared to be less than 10 years old. He only weighed 23 kg. He suffered from cystic fibrosis (CF). I saw him the first time, when I was on call in the Emergency room (ER) at King Faisal Specialist Hospital and Research Center (KFSHRC). Although he was severely malnourished, one could tell from his eyes, his manner of talking, and the confidence that oozed from him that he was a smart boy. He was suffering from an infective exacerbation, with fever, tachypnea, tachycardia and hypoxemia. When I told him that his condition necessitated hospital admission, it came as no surprise to him.

Fayez had recently been admitted to the local hospital in Qaseem, but as he had not been making any progress, he had discharged himself and had come with his older brother to Riyadh, getting himself admitted to the ER in our hospital.

Fayez made one plea. He requested to be admitted to a room where there was a window, so that he could see the sunlight. I told him that for the time being he needed to be admitted to the Intensive Care Unit (ICU), which had no windows. Realizing his advanced illness, and our inability to provide a cure that would end his suffering, I wished to fulfill his simple wish. I promised him that once his situation has improved, I would make every effort to transfer him to a room with a window.

I left Fayez under the care of one of my colleagues, who looked after him during his last admission. A week later, I was again on call and decided to check how Fayez was doing. He was still in the ICU. It was obvious that he was dying, despite maximal treatment, including ventilator support. By the time this article is published, Fayez would have died without seeing the sunlight, may Allah (God) grant his soul mercy and substitute him for his lost youth in this life with eternal youth in the Heavens.

Since Fayez was diagnosed with CF, he was seen in many hospitals in Saudi Arabia and abroad. He was initially treated in his local hospital, then referred to the National Guard Hospital in Riyadh, then Germany and finally to the KFSHRC. He was sent on an expensive trip to Germany for a few months, where he received treatment, including a controversial one – vaso-intestinal polypeptide (VIP). Despite this, his disease progressed relentlessly. This was probably due to multifactorial origin, including the condition being a severe form of CF and a number of social factors such as living away in small town, etc. However, I believe that one important factor was the lack of well-coordinated specialized respiratory care for his disease.

Thanks to these specialized units, the prognosis of CF has improved significantly over the last few decades, with more patients now being taken care of by adult physicians, rather than pediatricians.[1] These units employ a multi-disciplinary approach with well-trained personnel, including nurses, physiotherapists, nutritionists, social workers and psychologists, led by physicians interested in the care of CF.

Are there specialized units in Saudi Arabia to care for CF? I do not think so. In all tertiary care hospitals in Riyadh, for example, pediatric and adult pulmonologists will care for their own population, whether Ministry of Health, University, Military, National Guard or Security Forces. KFSHRC has the largest number of patients, but it has not received adequate support to set up a lead care center for CF. Occasionally there are cross referrals, but there is no organized way to overcome the logistic and administrative barriers. Sometimes, in the same hospital, each pulmonologist will have a handful of patients in his clinic.

The establishment of the Saudi Thoracic Society (STS), a number of years ago, was a major step in unifying the efforts of all physicians and other health professionals, for tackling challenges such as improving care of chronic respiratory diseases and bringing it to up-to-date standards. In these few years, the Society has made significant and noticeable strides and many achievements, the most recent one being the development of local guidelines to treat pulmonary arterial hypertension (PAH).[2] Despite this, however, each of the different sectors is treating very few patients with PAH, thereby hampering the development of experience and research. Interestingly, I received e-mails from different (government) centers offering to accept patients with PAH, since they had acquired some form of an inhalational prostaglandins, with the improvement in health budget this year and the vigorous promotional efforts of the manufacturers. Therefore, more effort is needed to overcome obstacles, resulting from the administrative divisions between the major health centers belonging to Ministry of Health, Military, National Guard, King Faisal Specialist Hospital, University and private hospitals.

There is still room for more coordination, cooperation and recognition for the need for specialization for each of these centers. Once this is achieved, the benefits for patients, education and research, and economy are enormous. Although the Council of Health Services has been established to serve this purpose, I am not aware of the steps being taken to facilitate this process. So, for the time being, it is up to us, individual health professionals and the STS, to exert more efforts to achieve this task and lobby for more support from the government and other agencies, to establish these specialized units.

Finally, my call is not only for cystic fibrosis, but also for many other difficult diseases, requiring multidisciplinary care and research. I mentioned PAH, but the list may include lung cancer, pulmonary rehabilitation, interstitial lung disease, multi-resistant tuberculosis etc.

The success of transplant units in Saudi Arabia is an example of how focusing efforts can be more productive. We look for the day when specialized respiratory units becomes another icon of health care in Saudi Arabia.
